# Does Trust Help to Improve Residents’ Perceptions of the Efficacy of Disaster Preparedness? Evidence from Wenchuan and Lushan Earthquakes in Sichuan Province, China

**DOI:** 10.3390/ijerph19084515

**Published:** 2022-04-08

**Authors:** Fengwan Zhang, Xueling Bao, Xin Deng, Wei Wang, Jiahao Song, Dingde Xu

**Affiliations:** 1 College of Management, Sichuan Agricultural University, Chengdu 611130, China; 2020309005@stu.sicau.edu.cn (F.Z.); 2020209052@stu.sicau.edu.cn (X.B.); wangwei@sicau.edu.cn (W.W.); songjiahao@sicau.edu.cn (J.S.); 2 College of Economics, Sichuan Agricultural University, Chengdu 611130, China; dengxin@sicau.edu.cn; 3 Sichuan Center for Rural Development Research, College of Management, Sichuan Agricultural University, Chengdu 611130, China

**Keywords:** trust, disaster preparedness, perceived efficacy, partial least squares structural equation model, earthquake-hit area

## Abstract

Disaster preparation can reduce the impact of an earthquake on residents. Residents are more likely to undertake disaster preparedness if they perceive it to be effective. However, few studies have analyzed the influence of trust on this perception. This study surveyed 327 households in areas stricken by the Wenchuan and Lushan earthquakes to explore these issues. Trust was divided into government trust, emotional trust, and social trust, while the efficacy of disaster preparedness was divided into self-efficacy and response efficacy. A partial least squares structural equation model was used to explore the influence of trust on perceptions of the efficacy of disaster preparedness. The results show that: (1) government trust can directly increase perceived efficacy and indirectly increase self-efficacy via emotional trust; (2) emotional trust can directly increase self-efficacy; (3) social trust can directly reduce self-efficacy while indirectly increasing it by increasing emotional trust. This study deepens our understanding of the relationship between trust and perceptions of the efficacy of disaster preparedness. This study can provide inspiration to improve risk communication and construct systems of community-based disaster-prevention.

## 1. Introduction

Natural hazards can be difficult to predict and very destructive, so they have always been a focus of human attention. In recent years, the losses caused by natural hazards have been increasing, posing unprecedented challenges to human life, productivity, safety, and development. Among all kinds of natural hazards, earthquakes are one of the most threatening to human beings [1]. China is located between the Pacific seismic belt and the Eurasian seismic belt, and earthquakes are frequent and severe. From 2000 to 2018, 214 earthquakes of magnitude 5 (the Richter scale) or above occurred in mainland China, causing direct economic losses of 1691.44 billion dollars [2]. It is worth noting that in recent years, China’s destructive earthquakes have mainly been concentrated in the western region, with Sichuan being the province with the most severe earthquake disasters [3]. With such frequent earthquakes and associated serious damage, reducing the losses for the residents in the event of a disaster has become a major challenge for governments and academia.

Many empirical studies have shown that reasonable disaster preparedness can effectively reduce the impact of disasters [4,5]. However, studies have also found that residents in many disaster-threatened areas were not fully prepared to avoid disaster [6,7,8]. For example, Atreya et al. [9] investigated the flood preparedness of communities in Mexico and found that communities with accessible flood risk maps shared flood experience with families, and shelters could enhance the possibility of family preparedness for action, but only 8% of people knew about the risk maps. Xu et al. [8] surveyed farmers in areas prone to landslides in southwestern China also found that, in response to natural hazards such as landslides, 67% of the households did not have disaster prevention behaviors, and the farmers’ awareness of disaster prevention was relatively low. Therefore, many studies have tried to reveal the factors affecting the disaster preparedness of residents from different perspectives [10,11,12]. In fact, whether individuals or families adopt disaster preparedness depends on their subjective assessment of risk and coping choices. Self-efficacy and response efficacy are two of the most powerful predictors of risk-mitigation behavior [13,14,15]. Some studies have pointed out that improving self-efficacy and response efficacy will encourage people to take self-protection measures [16,17,18]. Self-efficacy refers to an individual’s belief in their ability to carry out the course of action required for a specific achievement [19]. Response efficacy refers to the extent to which individuals believe that protective measures can effectively reduce risks [20]. Kievik et al. [13] integrated the two into the concept of perceived efficacy. They pointed out that low perceived efficacy in residents of flood-prone areas occurred when they did not know whether they had the ability to carry out actions that might reduce the threat of floods and were uncertain of whether such actions were effective. When residents think things like, “I can deal with the threat” and “I know how to deal with the threat and be useful”, they will consciously consider ways to deal with the threat. That is, perceived efficacy is an important factor motivating residents to be prepared. In addition, perceived efficacy also plays an important role in the theory of protective motivation [21,22]. Protective motivation theory links an individual’s intention to take protective measures with threat assessment and coping assessment [23]. Among them, coping assessment factors include individual self-efficacy, protective response efficacy, and response cost. The importance of response assessment for the intention to take measures to mitigate natural hazards has been emphasized by many scholars, although the actual behavior of residents may remain conservative due to cost [24,25].

The theory of planned behavior (TPB) holds that intention is the central factor in the performance of specific behaviors [26]. Risk communication can effectively stimulate the intention of residents to seek information and self-protection [13]. Steelman and Mccaffrey [27] studied fire cases in three regions and proposed that effective information communication is the key practice to achieve the goal of better disaster resistance. In the process of risk information transmission, trust is the core mechanism that simplifies complexity and reduces uncertainty [28], and its different components are important factors that affect the willingness of people to prepare for disaster avoidance or behavioral decision-making [29,30]. According to existing studies, residents’ trust in different sources of information does not play the same role in risk management. Steelman et al. [31] divided disaster information sources into family/friends/neighbors, mass media, and official sources, and using data from five fires, found that most respondents used information sources when faced with fires that were different from what they considered useful. Kirschenbaum et al. [32] used the preparedness data from residents in the Israeli earthquake zone and found that trust in both formal and informal sources of information had a significant impact on actual and perceived readiness. Lin et al. [33] proposed that the residents’ trust in the government, experts, and the media is a positive predictor of disaster-mitigation willingness in victims of flood and landslide. In addition, reduced trust in public risk measures and the associated lack of personal responsibility have also been shown to be inversely correlated with risk mitigation intentions [34,35]. However, Terpstra [36], through an investigation of a Dutch flood disaster, pointed out that trust in the government reduces individual willingness to take preventive measures. At the same time, studies on public flood-risk cognition and prevention in mainland China show that trust reduces the tendency for long-term flood-control preparation [37].

In the face of disaster, residents will theoretically take reasonable disaster preparedness measures to minimize risks and maximize benefits. So, is the low actual disaster preparedness of residents related to perceived efficacy or trust? What are the different effects of the residents’ trust in different sources of information on their perceived efficacy? There seems to be little research that can answer these questions. In this context, this paper studied 327 households in the Wenchuan and Lushan earthquake disaster areas. A partial-least-squares structural equation model (PLS-SEM) was used to explore the relationship between the residents’ perceived efficacy and trust in order to provide a reference that can help improve the willingness of residents to avoid disasters and help the government formulate policies related to disaster prevention and mitigation. It is noted that although our findings are specific to the Sichuan earthquake threat area, they may provide ideas for disaster prevention and mitigation in other areas, such as community risk communication, etc. Compared with previous studies, this study provides the following marginal contributions: firstly, it tries to construct a theoretical analysis framework for trust and perceived efficacy of disaster preparedness. This framework has a stronger explanatory power for the real world, which is conducive to enriching the understanding of disaster communication in theory and practice. Secondly, compared with using only a Logit or Probit model (as in past studies), PLS-SEM has stronger applicability and explanatory power, and can better reflect the interactions between various factors.

The subsequent structure of this paper is as follows: the second section contains our theoretical analysis and research hypotheses; the third section introduces the data and methods used in this paper; the fourth section empirically tests the influence of trust on perceived efficacy; the fifth section discusses the results; and the sixth section summarizes the whole paper.

## 2. Theoretical Analysis and Research Hypothesis

The decision-making model of conservation action is a multi-stage model for individuals making decisions about imminent or future threats in order to prevent environmental hazards. The core idea of the pre-decision stage is that environmental cues, social cues, and socially transmitted warnings can affect the public’s core perception of environmental threats, alternative conservation actions, and stakeholders, thus providing a basis for protective behavior decision-making [38]. Based on this idea, this study explores the correlation between the trust in risk communication and the perceived efficacy of disaster preparedness. Theoretically, perceived efficacy determines whether people have the motivation to control danger or the fear of a threat [13]. When people believe they can respond effectively to a threat, they are motivated to control the risk and consider ways to eliminate or mitigate the threat. In this case, people will carefully consider measures recommended in persuasive messages as a means of controlling the risk. Therefore, this study hypothesizes that trust in information from different sources will have different effects on the perceived efficacy of residents. The specific action path is as follows.

Firstly, the government, as the official channel, has more specialized disaster knowledge and access to scientific information than the public. Therefore, if governmental information about disasters is trusted by residents, the cost of searching for information and their fear of uncertainty can be reduced [7,39,40]. Therefore, if residents have enough trust in the government, they will be willing to follow government advice to take preparatory actions and will have a higher degree of belief in the efficacy and practicality of preventing private damage. Secondly, in addition to trust in the government’s formal social network, the transmission of risk information and disaster-related knowledge through word-of-mouth and informal social relations is also powerful [32]. Studies have found that help from family members and neighbors is a major source of social support when people are coping with an emergency, which will inevitably affect public opinion and actual preventive behavior [41,42]. Therefore, trust in informal social networks is also a feasible way to enhance the perceived efficacy of residents. On the one hand, a relationship circle based on emotional trust can provide residents with materials, resources, and emotional support [43]. Trust in a familiar network of relatives and friends will make up for deficiencies in their own knowledge and skills, and expand access to information in the way of, “the fire is high when everyone adds firepower”, and realize, “the more truth is discerned, the clearer it is”, thus improving the residents’ perceived efficacy. On the other hand, for social trust, word-of-mouth may positively stimulate perceived efficacy in the way that, “it is better to believe it than not to believe it”. However, earthquakes, as natural hazards occurring in a century, are extremely rare. Under the psychological effect of survivors’ bias, the residents of the hardest-hit areas will think: the possibility of encountering another big earthquake is very low; underestimate the possibility of disaster losses; and that disaster preparedness can be reduced or even eliminated. According to the principle of survivor bias, this study is more inclined to the latter. That is, it is assumed that social trust is negatively correlated with the residents’ perceived efficacy.

Information dissemination is rarely smooth at the beginning of a disaster and information channels may be varied and indirect. Therefore, the residents’ trust in different information sources may have an indirect mediating effect on their perceived efficacy. Firstly, residents may get official information released by the government through their relatives and friends. The authority of government information, and consistency with the goals of relatives and friends, can effectively increase the residents’ perceived efficacy. On the other hand, although messages from strangers may have positive or negative impacts on the residents’ perceived efficacy, it can also have a positive impact if it is delivered through emotional trust. Secondly, the residents’ trust in the government can also indirectly influence social trust and, thus, affect the residents’ perceived efficacy. However, since governmental information can effectively break the uncertainty of information, this study also believes that it is positively correlated with the residents’ perceived efficacy.

To sum up, based on the conclusions and theoretical analyses from the existing literature, the following hypotheses are made related to the relationship between trust and the perceived efficacy of residents in earthquake-hit areas (the theoretical model is shown in Table 1):

**H1.**  *Government trust is directly and positively correlated with self-efficacy*.

**H1a.**  *Government trust is indirectly and positively correlated with self-efficacy through the influence of affective trust*.

**H1b.**  *Government trust is indirectly and positively correlated with self-efficacy through the influence of social trust*.

**H1c.**  *Government trust indirectly influences the residents’ emotional trust by positively influencing social trust, and finally has a positive and significant correlation with the residents’ self-efficacy*.

**H2.**  *Government trust is directly and positively correlated with response efficiency*.

**H2a.**  *Government trust is indirectly and positively correlated with response efficacy through the influence of affective trust*.

**H2b.**  *Government trust is indirectly and positively correlated with response efficacy through the influence of social trust*.

**H2c.**  *Government trust indirectly affects the residents’ emotional trust by positively influencing social trust, and ultimately has a positive and significant correlation with the residents’ response efficiency*.

**H3.**  *Emotional trust is directly and positively correlated with self-efficacy*.

**H4.**  *Emotional trust is directly and positively correlated with response efficacy*.

**H5.**  *Social trust is directly and negatively correlated with self-efficacy.*

**H5a.**  *Social trust is indirectly and positively correlated with self-efficacy by influencing affective trust.*

**H6.**  *Social trust is directly and negatively correlated with response efficacy*.

**H6a.**  *Social trust is indirectly and positively correlated with response efficacy through the influence of affective trust*.

**H7.**  *Government trust is positively and significantly correlated with affective trust*.

**H8.**  *Social trust is positively and significantly correlated with affective trust.*

**H9.**  *Government trust is positively and significantly correlated with social trust.*

**H9a.**  *Government trust is positively and significantly correlated with affective trust and the influence of social trust.*

## 3. Materials and Methods

### 3.1. Data Collection

**Figure 1:**
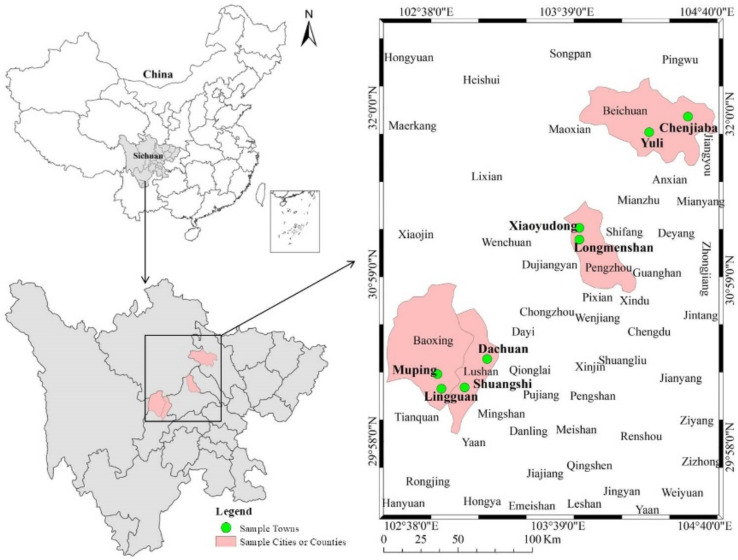
Location map of sample counties and towns.

Sichuan Province is one of the worst earthquake-disaster-affected regions of China and even the world. The 2008 Wenchuan earthquake (8 on the Richter scale) and the 2013 Lushan earthquake (7 on the Richter scale) are the two earthquakes that have caused the greatest losses of life and property in China this century, causing 446,000 casualties and 13,200 casualties and direct economic losses of 134.69 billion dollars and 10.46 billion dollars, respectively [44,45]. The data in this paper were obtained from a questionnaire survey of 327 households in the areas worst-hit by the Wenchuan and Lushan earthquakes that was conducted by our research group in July 2019. The main content of this survey involved disaster risk perception and disaster avoidance behavior response. The survey method involved one-on-one household interviews by investigators, and each questionnaire took 1–1.5 h. In total, 327 valid questionnaires were obtained from 4 districts, 8 towns and 16 villages. In order to ensure the typicality and representativeness of the survey, stratified probabilistic random sampling was adopted to determine the survey samples. For the specific sampling process, see Xu et al. [46]. It is worth mentioning that, considering that the study sample districts should be from the disaster-stricken areas of the Wenchuan and Lushan earthquakes, care was taken to select at least two districts with significant differences in economic development for each area. In this study, Beichuan and Pengzhou were selected as the sample counties from the 10 counties severely affected by the Wenchuan earthquake (Wenchuan, Maoxian, Beichuan, Qiang Minority Autonomous County, Anxian, Pingwu, Mianzhu, Shifang, Dujiangyan, Pengzhou, and Qingchuan). From the six counties in the Lushan earthquake disaster area (Lushan, Yucheng District, Tianquan, Mingshan, Yingjing and Baoxing), Baoxing and Lushan were selected as the sample counties, so it can be considered that this survey has good representation. Figure 1 shows a location map of the sample counties and towns.

### 3.2. Variable Measurement

The core independent variable of this study was trust. Referencing the definition and measure of trust proposed by Xue K.J et al. [47], Menozzi and Finardi [48], Zhao et al. [43], Welter [49], Han et al. [50], and Paton et al. [51], this paper defines trust as: the truster holds a positive evaluation of the trusted one, believes that the behavior of the trusted one is beneficial to the truster, and the truster will make corresponding behavior under the guidance of this psychological attitude. A high level of trust not only exists in friendly interpersonal communication, but also forms in a harmonious organizational relationship. According to the different information sources, this study divided trust into government trust, emotional trust, and social trust, and designed 5-level Likert scales to measure each of them (see Table 2 for details). Among them, government trust was measured as the residents’ trust in governmental disaster judgment, government decision-making, and the government overall. The object of emotional trust is usually the people that are interacted with, such as relatively close friends and family members. Social trust refers to the wise and reciprocal behavior, when necessary, during social interaction [52]. It is measured by the residents’ degree of trust in the verbal information they receive.

The dependent variable in this study was perceived efficacy. According to the research of Xue K.J et al. [47], Grothmann et al. [23], Kievik et al. [13], and Mertens et al. [21], we divided perceived efficacy into self-efficacy and response efficacy, which were each measured with 5-level Likert scales (Table 2). Among them, self-efficacy is an individual’s perceived ability to implement protective responses. In this paper, the residents’ understanding of evacuation routes, locations of emergency shelters, and disaster prevention and mitigation measures were measured. Response efficacy is related to the judgment of whether protective behavior will be effective in protecting oneself or others from harm. Evacuation, a common disaster avoidance behavior, is a method to prepare people for an imminent threat [53,54], which can effectively weaken the impact of disasters. Therefore, response efficacy was measured by the degree to which residents agreed that evacuation would effectively prevent injury, death, and suffering.

### 3.3. Research Methods

In view of the exploratory nature of this study, the small sample size, and the existence of formative indicators, this study mainly uses the partial least squares (PLS) method to conduct empirical analysis of the measurement model using Smart PLS 3.0 software (This software is released by SmartPLS GmbH (Ismaning, Germany) for data analysis based on PLS-SEM method). Compared with other structural equation models based on covariance methods, PLS has no preset data distribution, and there is no need to test whether the data conform to the assumption of normal distribution. At the same time, it can predict the weights and loads of all indicators and causal relationships in the multi-stage model [55,56], which is very effective for identifying multiple key target structures and their most important influencing factors, and is suitable for exploratory and explanatory studies.

## 4. Results

### 4.1. Sample Characteristics

According to the preliminary statistics for the questionnaire, among 327 resident samples, the average age of the respondents was 53.41 years old, and males accounted for 54%. The education level of respondents was generally low, with an average of 6.29 years of education. The average household size was 4.13 people, and the annual cash income was 10,252.08 dollars.

As shown in Table 2, among the core variables, the mean values for government trust, emotional trust, social trust, self-efficacy, and response efficiency were 4.37, 4.35, 2.74, 3.81, and 4.33, respectively. This shows that residents trust the government most, followed by family and friends, and finally the public. The differences in trust and perceived efficacy for different groups were further analyzed according to gender and age, as shown in Table 3. In terms of gender, the mean values for government trust (4.43), self-efficacy (3.92), and response efficacy (4.35) for males were higher than those for females (4.30, 3.69 and 4.29, respectively), but social trust (2.63) was lower than that for females (2.86). The mean values for emotional trust were the same for males and females (4.35). In terms of age, referring to the research of He and Zhang [57], farmers were divided into the new generation (born after 1980) and the old generation (born before 1980). The mean values for government trust (4.23), emotional trust (4.24), social trust (2.60), and response efficacy (4.32) for the new generation were lower than those for the old generation (4.39, 4.37, 2.76 and 4.33, respectively), and the mean value for self-efficacy (4.22) was higher for the new generation than the old generation (3.75).

### 4.2. Reliability and Validity Tests of External Models

#### 4.2.1. Reliability Tests

Reliability tests are used to verify the consistency and reliability of data. In this study, the reliability of the model was evaluated with two metrics: Cronbach’s alpha and composite reliability (CR). As shown in Table 4, Cronbach’s alpha for the five potential variables of government trust, emotional trust, social trust, self-efficacy, and response efficacy were all greater than 0.6 (0.701, 0.655, 0.805, 0.645 and 0.799, respectively). At the same time, CR values were all over 0.7 (0.834, 0.852, 0.910, 0.808 and 0.880, respectively), indicating that the entries designed for the study had good reliability.

#### 4.2.2. Validity Tests

Besides reliability tests, validity tests were also needed for each dimension. As shown in Table 4, the standardized load of the reliability of a single project was evaluated in this study, and the factor load for all latent variables in this study reached the ideal value of 0.7. The average variance extracted (AVE) was used to measure the aggregation validity of each dimension. The AVEs for all variables in this study were greater than 0.5, indicating that each dimension had good aggregation validity. In addition, discriminant validity was determined by the Fornell-Larcker criterion [58]. As shown in Table 5, the diagonal values (bold) are larger than the values in the rows and columns, indicating that each dimension had discriminant validity.

#### 4.2.3. Multicollinearity Tests

In this study, the existence of multicollinearity among the variables was tested. The results show that the variance inflation factors (VIF) for the models were all less than 3, indicating that there was no serious collinearity between the variables.

### 4.3. Internal Model Test Results

The internal model test focuses on the value of the path coefficient, which explains the total effect of the interaction between the underlying variables. In this study, the bootstrap method was used to estimate the standard deviation of the parameters to be estimated, and *t*-values were used to determine whether the causal relationships between variables were significant (*t* > 1.96 means the parameter to be estimated is significant at the significance level of 0.05). The model estimation results are shown in Table 6.

In terms of direct influence, H1 and H2 were supported, such that government trust was positively and significantly correlated with self-efficacy and response efficacy with path coefficients of 0.143 and 0.246, respectively. This indicates that when other conditions remain unchanged, increasing one unit of government trust will increase self-efficacy by 0.144 units and response efficacy by 0.244 units on average. At the same time, emotional trust was positively and significantly correlated with self-efficacy, and its path coefficient was 0.215, indicating that increasing affective trust by one unit will increase self-efficacy by 0.215 units. Hypothesis H3 was supported; in addition, there was a significant negative correlation between social trust and self-efficacy. A one-unit increase in social trust will decrease self-efficacy by 0.176 units. Hypothesis H5 was supported. In terms of the interaction between the dimensions of trust, government trust and social trust were significantly correlated with emotional trust, and the impact of the size of emotional trust size were basically the same. The path coefficients were 0.187 and 0.185, namely, one additional unit of government trust and social trust will increase emotional trust by 0.187 and 0.185 units, respectively.

In terms of indirect influences, there were two obvious paths, (1) government trust→ emotional trust → self-efficacy, and (2) social trust → emotional trust → self-efficacy. The path coefficients were both 0.040, which means that H1a and H5a were supported. The specific influence process is as follows: increasing one unit of government trust will increase 0.187 units of emotional trust; and increasing one unit of emotional trust will increase 0.215 units of self-efficacy. Therefore, increasing one unit of government trust will indirectly increase self-efficacy by 0.040 units. Similarly, increasing social trust by one unit increases affective trust by 0.187 units, which then increases self-efficacy by 0.040 units.

## 5. Discussion

In general, among the core latent variables in this paper, the residents trusted the government most, followed by family and friends, and finally the public. The residents’ perception of response efficiency was also stronger than that of self-efficacy. Although gender and age groups had different perceptions of the core variables, overall, the differences were not significant.

The three trust variables considered in this paper were directly or indirectly related to perceived efficacy. In terms of self-efficacy, the residents’ trust in the government and familiar social networks (family members, relatives, and friends) can directly promote the improvement of self-efficacy, and government trust can have a positive impact on emotional trust, and then indirectly, have a positive impact on self-efficacy. Government trust reflects the people’s confidence in the government’s abilities and information, while emotional trust reflects harmonious interpersonal relationships and a higher degree of cooperation in the neighborhood. The higher the level of government trust and emotional trust that residents have, the more frequent the communication between the two will be. In this way, residents will have easier access to disaster-related information, which could influence residents to rely on others to share resources, resolve conflicts, and continue cooperation in faith to eventually increase the chances of disaster adaptation. It is worth mentioning that although government trust and emotional trust were both positively correlated with self-efficacy, emotional trust had a greater impact than government trust, indicating that residents are most susceptible to the influence of intimate relationship circles. This is consistent with the study by Steelman et al. [31]. In addition, the residents’ trust in unfamiliar positive or negative information had a direct and significant negative correlation with self-efficacy. It can be indirectly and positively correlated with self-efficacy via a positive impact on emotional trust, but the effect is small. Social trust had an overall negative and significant impact on self-efficacy. In addition to survivor bias, this may be explained by experience. Experience may reduce the level of preparation [59]. For residents who have risk experience, especially two earthquakes with a magnitude greater than 7, they think they have handled it well in the past, which makes them think they can cope with future events without having to prepare for a disaster, and therefore, have lower self-efficacy. Furthermore, from the perspective of psychology, when people trust each other, they will be less defensive and confrontational; that is to say, residents in a trustful environment are more likely to make a positive decision [60]. However, the authenticity of widespread verbal messages is unknown, and the occurrence of an earthquake is a typical uncertain event. Faced with such news, people have a high level of resistance, are reluctant to admit that they are in danger, are prone to make negative decisions, and avoid participating in preventive activities.

As far as response efficacy is concerned, only government trust had a direct positive correlation with response efficacy, while affective trust and social trust did not. The reason for this may be that when people are unsure about whether their response will be effective, i.e., when they are unsure whether their actions can alleviate a disaster threat, they will have a sense of powerlessness [33]. That is, they may have a fatalistic attitude when faced with a dangerous or catastrophic situation where people are not prepared to avoid disaster. Compared with trust in informal networks, government trust is authoritative. The information and measures issued by authorities can effectively prevent residents from feeling powerless and effectively improve their response efficiency. At the same time, risk-adjustment properties are important in predicting the act of preparing [39,61,62,63,64]. In this study, response efficacy was measured by assessing the belief that residents felt that they were safe from danger by evacuating during an earthquake. Although evacuation is a very effective way to save the lives, other coping behaviors, like relocation and emergency preparedness, may lead to the deviation of the residents’ response to perceived results [65,66,67,68,69].

Compared with other countries, China has a vast area of mountainous terrain and a frequent earthquake hazard, so it is of great significance to explore the residents’ perception of the efficacy of disaster preparedness. Based on the above analysis, this study makes three suggestions to improve residents’ awareness and willingness to prepare for disasters. First, in terms of risk communication channels, the government should strengthen awareness of disaster risk-management systems via the media and public opinion to enhance the residents’ social trust level, especially for rural residents. In addition, China’s current disaster-prevention system is community-based. In this context, the residents’ trust in the government is largely reflected in their trust for community management organizations. Therefore, it is necessary to strengthen the direct risk communication between community managers and community residents. Moreover, it is also an effective way to enhance the perceived effectiveness of the residents’ disaster preparedness by strengthening cooperation and communication among family members, friends, and neighbors in the community. For example, the government and the community can strengthen communication between community and family, and among family members, by organizing family-oriented escape drills and knowledge popularization activities so as to enhance the level of trust among different subjects and realize an improvement in the perceived effectiveness of disaster avoidance. It is worth noting that with the continuous development of big data in recent years, the role of the Internet in promoting disaster preparedness cannot be ignored [70,71]. According to the 49th Statistical Report on Internet Development in China, by December 2021, the number of Internet users in rural China had increased to 284 million, and the Internet penetration rate had increased to 57.6%. It can be said that the popularization and development of informatization has greatly changed the way of life for Chinese farmers. Therefore, different information sources can use the Internet to carry out targeted activities. For example, the government can use search data from websites such as Baidu to assess the impact of disaster information on the users’ interests. The community and public media can also carry out targeted advertising campaigns on the Internet in a timely manner to improve the perceived effectiveness of the public, thereby promoting the adoption of preventive measures. Second, in terms of the content of risk communication, besides perceptions of danger, government or community organizations also need to understand the residents’ perceptions of good risk adjustments. Namely, in addition to strengthening construction of the government’s disaster prevention system and publicity for disaster prevention knowledge and measures, more emphasis should be placed on whether the efficacy of these knowledge and action measures is meaningful. By introducing effective and ineffective coping strategies to residents at the same time, the residents’ perceived effectiveness can be improved and their actual coping actions to avoid disasters can be finally affected. Third, in terms of risk communication strategies, any goal orientation should come with achieving goals. For example, asking residents to prepare emergency equipment or learn relevant skills and knowledge will give them experience in correct and useful techniques that will enhance their perception of their efficacy.

## 6. Conclusions, Limitations, and Perspectives

Using survey data from 327 farmers in areas affected by the Wenchuan earthquake and Lushan earthquake in Sichuan Province, a partial least squares structural equation model system was used to explore the influence of trust on the perceived effectiveness of the residents’ disaster preparedness. The results showed the following:

(1) Government trust can directly increase perceived efficacy and indirectly increase self-efficacy via emotional trust.
(2) Emotional trust can directly increase self-efficacy.
(3) Social trust can directly reduce self-efficacy while indirectly increasing it by increasing emotional trust.

Although this study has made some beneficial advances and obtained some important results, there are still some deficiencies. Firstly, this study only focused on the correlation between the residents’ trust and the perceived effectiveness of disaster avoidance in the Sichuan earthquake threat area. Future studies can further explore the correlation between trust and perceived effectiveness and the residents’ actual disaster avoidance preparedness. Secondly, this study only selected peasant households in the Wenchuan and Lushan earthquake disaster areas, and it remains to be explored whether the research results can be applied to places with different geographical characteristics.

## Figures and Tables

**Table 1:** Theoretical hypothesis table.

Hypothesis	Path	Hypothetical Direction
H1	Government trust -> Self-efficacy	Positive
H2	Government trust -> Response efficacy	Positive
H1a	Government trust -> Emotional trust -> Self-efficacy	Positive
H2a	Government trust -> Emotional trust -> Response efficacy	Positive
H1b	Government trust -> Social trust -> Self-efficacy	Positive
H2b	Government trust -> Social trust -> Response efficacy	Positive
H1c	Government trust -> Social trust -> Emotional trust -> Self-efficacy	Positive
H2c	Government trust -> Social trust -> Emotional trust -> Response efficacy	Positive
H3	Emotional trust -> Self-efficacy	Positive
H4	Emotional trust -> Response efficacy	Positive
H5	Social trust -> Self-efficacy	Negative
H6	Social trust -> Response efficacy	Negative
H5a	Social trust -> Emotional trust -> Self-efficacy	Positive
H6a	Social trust -> Emotional trust -> Response efficacy	Positive
H7	Government trust -> Emotional trust	Positive
H8	Government trust -> Social trust	Positive
H9	Social trust -> Emotional trust	Positive
H9a	Government trust -> Social trust -> Emotional trust	Positive

**Table 2:** Definition and descriptive statistics of the variables in the mode.

Category	Latent Variable	Item Description	Code	Mean Value	Standard Deviation
Trust	Government trust	How much trust do you have in the government’s judgment of disasters? ^a^	GT1	4.37	0.821
		People in the village have great faith in the government’s decisions. Do you agree? ^a^	GT2	4.28	0.876
		In general, your level of trust in government ^a^	GT3	4.46	0.838
	Emotional trust	In general, how much do you trust your friends and family? ^a^	AT1	4.05	0.912
		In general, what is your level of trust in your family? ^a^	AT2	4.65	0.760
	Social trust	Do you feel credibility in the positive verbal messages you receive? ^a^	ST1	2.91	1.224
		Do you feel credibility in the negative verbal messages you receive? ^a^	ST2	2.56	1.222
Perceived efficacy	Self-efficacy	When a disaster occurs, do you know the evacuation route? ^b^	SE1	4.17	1.174
		Do you know the locations of emergency shelters in the village? ^b^	SE2	3.98	1.278
		Do you know the appropriate disaster-prevention and mitigation measures for the village? ^b^	SE3	3.28	1.308
	Response efficacy	Can evacuation effectively prevent injury/death? ^b^	CE1	4.37	0.879
		Can evacuation effectively reduce pain? ^b^	CE2	4.28	0.914
		If I evacuate, will I effectively avoid injury/death? ^b^	CE3	4.33	0.908

Note: ^a^ 5-level Likert scale, 1 represents great distrust and 5 represents great trust; ^b^ 5-level Likert scale, 1 represents great distrust and 5 represents great trust.

**Table 3:** Statistical tables of core variables for different ages and genders.

Category	Latent Variable	Code	Gender		Age	
			Man	Woman	New Generation	Old Generation
			Mean Value	Mean Value	Mean Value	Mean Value
Trust	Government trust	GT1	4.43	4.30	4.31	4.38
		GT2	4.35	4.20	4.12	4.31
		GT3	4.50	4.40	4.26	4.48
	Emotional trust	AT1	4.06	4.03	3.85	4.07
		AT2	4.64	4.66	4.62	4.66
	Social trust	ST1	2.77	3.06	2.62	2.95
		ST2	2.48	2.66	2.57	2.56
Perceived efficacy	Self-efficacy	SE1	4.29	4.04	4.67	4.10
		SE2	4.09	3.85	4.26	3.94
		SE3	3.38	3.17	3.74	3.21
	Response efficacy	CE1	4.37	4.36	4.40	4.36
		CE2	4.39	4.27	4.31	4.34
		CE3	4.30	4.25	4.26	4.28

**Table 4:** Validity and reliability analysis of the measurement model.

Latent Variables	Code	Factor Loading	Cronbach’s Alpha	CR	AVE
Government trust	GT1	0.770	0.701	0.833	0.625
	GT2	0.774			
	GT3	0.826			
Emotional trust	AT1	0.870	0.655	0.853	0.744
	AT2	0.854			
Social trust	ST1	0.926	0.805	0.911	0.836
	ST2	0.903			
Self-efficacy	SE1	0.738	0.645	0.808	0.584
	SE2	0.778			
	SE3	0.777			
Response efficacy	CE1	0.834	0.799	0.881	0.713

**Table 5:** Pearson correlation matrix and average extraction.

Latent Variables	Response Efficacy	Emotional Trust	Government Trust	Social Trust	Self-Efficacy
Response efficacy	**0.844**				
Emotional trust	0.067	**0.862**			
Government trust	0.249	0.195	**0.790**		
Social trust	−0.040	0.194	0.046	**0.915**	
Self-efficacy	0.292	0.209	0.177	−0.128	**0.764**

**Table 6:** Description of model hypothesis test results.

Hypothesis	Path	Coefficient	*t* Value	*p* Value	The Inspection Results
H1	Government trust → Self-efficacy	0.143 **	2.470	0.014	Support
H2	Government trust → Response efficacy	0.246 ***	4.243	0.000	Support
H1a	Government trust → Emotional trust → Self-efficacy	0.040 **	2.451	0.014	Support
H2a	Government trust → Emotional trust → Response efficacy	0.006	0.430	0.667	Nonsupport
H1b	Government trust → Social trust → Self-efficacy	−0.008	0.640	0.523	Nonsupport
H2b	Government trust → Social trust → Response efficacy	−0.003	0.395	0.693	Nonsupport
H1c	Government trust → Social trust → Emotional trust → Self-efficacy	0.002	0.617	0.537	Nonsupport
H2c	Government trust → Social trust → Emotional trust → Response efficacy	0.000	0.245	0.807	Nonsupport
H3	Emotional trust → Self-efficacy	0.215 ***	3.604	0.000	Support
H4	Emotional trust → Response efficacy	0.030	0.465	0.642	Nonsupport
H5	Social trust → Self-efficacy	−0.176 **	2.690	0.007	Support
H6	Social trust → Response efficacy	−0.057	0.812	0.417	Nonsupport
H5a	Social trust → Emotional trust → Self-efficacy	0.040 **	2.577	0.010	Support
H6a	Social trust → Emotional trust → Response efficacy	0.006	0.454	0.650	Nonsupport
H7	Government trust → Emotional trust	0.187 **	3.299	0.001	Support
H8	Government trust → Social trust	0.046	0.689	0.491	Nonsupport
H9	Social trust → Emotional trust	0.185 ***	3.785	0.000	Support
H9a	Government trust → Social trust → Emotional trust	0.008	0.662	0.508	Nonsupport

Note: ** means *p* < 0.05, and *** means *p* < 0.01.

## Data Availability

If necessary, we can provide raw data.
